# The Relation of Violent Video Games to Adolescent Aggression: An Examination of Moderated Mediation Effect

**DOI:** 10.3389/fpsyg.2019.00384

**Published:** 2019-02-21

**Authors:** Rong Shao, Yunqiang Wang

**Affiliations:** ^1^ Research Institute of Moral Education, College of Psychology, Nanjing Normal University, Nanjing, China; ^2^ The Lab of Mental Health and Social Adaptation, Faculty of Psychology, Research Center for Mental Health Education, Southwest University, Chongqing, China

**Keywords:** violence video games, aggression, family environment, normative beliefs about aggression, moderated mediation effect

## Abstract

To assess the moderated mediation effect of normative beliefs about aggression and family environment on exposure to violent video games and adolescent aggression, the subjects self-reported their exposure to violent video games, family environment, normative beliefs about aggression, and aggressive behavior. The results showed that there was a significant positive correlation between exposure to violent video games and adolescent aggression; normative beliefs about aggression had a mediation effect on exposure to violent video games and adolescent aggression, while family environment moderated the first part of the mediation process. For individuals with a good family environment, exposure to violent video games had only a direct effect on aggression; however, for those with poor family environment, it had both direct and indirect effects mediated by normative beliefs about aggression. This moderated mediation model includes some notions of General Aggression Model (GAM) and Catalyst Model (CM), which helps shed light on the complex mechanism of violent video games influencing adolescent aggression.

## Introduction

### Violent Video Games and Aggression

The relationship between violent video games and adolescent aggression has become a hot issue in psychological research (Wiegman and Schie, 1998; Anderson and Bushman, 2001; Anderson et al., 2010; Ferguson et al., 2012; Greitemeyer, 2014; Yang et al., 2014; Boxer et al., 2015). Based on the General Aggression Model (GAM), Anderson et al. suggested that violent video games constitute an antecedent variable of aggressive behavior, i.e., the degree of exposure to violent video games directly leads to an increase of aggression (Anderson and Bushman, 2001; Bushman and Anderson, 2002; Anderson, 2004; Anderson et al., 2004). Related longitudinal studies (Anderson et al., 2008), meta-analyses (Anderson et al., 2010; Greitemeyer and Mugge, 2014), event-related potential studies (Bailey et al., 2011; Liu et al., 2015), and trials about juvenile delinquents (DeLisi et al., 2013) showed that exposure to violent video games significantly predicts adolescent aggression.

Although Anderson et al. insisted on using the GAM to explain the effect of violent video games on aggression, other researchers have proposed alternative points of view. For example, a meta-analysis by Sherry (2001) suggested that violent video games have minor influence on adolescent aggression. Meanwhile, Ferguson (2007) proposed that publication bias (or file drawer effect) may have implications in the effect of violent video games on adolescent aggression. Publication bias means that compared with articles with negative results, those presenting positive results (such as statistical significance) are more likely to be published (Rosenthal and Rosnow, 1991). A meta-analysis by Ferguson (2007) found that after publication bias adjustment, the related studies cannot support the hypothesis that violent video games are highly correlated with aggression. Then, Ferguson et al. proposed a Catalyst Model (CM), which is opposite to the GAM. According to this model, genetic predisposition can lead to an aggressive child temperament and aggressive adult personality. Individuals who have an aggressive temperament or an aggressive personality are more likely to produce violent behavior during times of environmental strain. Environmental factors act as catalysts for violent acts for an individual who have a violence-prone personality. This means that although the environment does not cause violent behavior, but it can moderate the causal influence of biology on violence. The CM model suggested that exposure to violent video games is not an antecedent variable of aggressive behavior, but only acts as a catalyst influencing its form (Ferguson et al., 2008). Much of studies (Ferguson et al., 2009, 2012; Ferguson, 2013, 2015; Furuya-Kanamori and Doi, 2016; Huesmann et al., 2017) found that adolescent aggression cannot be predicted by the exposure to violent video games, but it is closely related to antisocial personality traits, peer influence, and family violence.

Anderson and his collaborators (Groves et al., 2014; Kepes et al., 2017) suggested there were major methodological shortcomings in the studies of Ferguson et al. and redeclared the validity of their own researches. Some researchers supported Anderson et al. and criticized Ferguson’s view (Gentile, 2015; Rothstein and Bushman, 2015). However, Markey (2015) held a neutral position that extreme views should not be taken in the relationship between violent video games and aggression.

In fact, the relation of violent video games to aggression is complicated. Besides the controversy between the above two models about whether there is an influence, other studies explored the role of internal factors such as normative belief about aggression and external factors such as family environment in the relationship between violent video games and aggression.

### Normative Beliefs About Aggression, Violence Video Games, and Aggression

Normative beliefs about aggression are one of the most important cognitive factors influencing adolescent aggression; they refer to an assessment of aggression acceptability by an individual (Huesmann and Guerra, 1997). They can be divided into two types: general beliefs and retaliatory beliefs. The former means a general view about aggression, while the latter reflects aggressive beliefs in provocative situations. Normative beliefs about aggression reflect the degree acceptance of aggression, which affects the choice of aggressive behavior.

Studies found that normative beliefs about aggression are directly related to aggression. First, self-reported aggression is significantly correlated to normative beliefs about aggression (Bailey and Ostrov, 2008; Li et al., 2015). General normative beliefs about aggression can predict young people’s physical, verbal, and indirect aggression (Lim and Ang, 2009); retaliatory normative beliefs about aggression can anticipate adolescent retaliation behavior after 1 year (Werner and Hill, 2010; Krahe and Busching, 2014). There is a longitudinal temporal association of normative beliefs about aggression with aggression (Krahe and Busching, 2014). Normative beliefs about aggression are significantly positively related to online aggressive behavior (Wright and Li, 2013), which is the most important determining factor of adolescent cyberbullying (Kowalski et al., 2014). Teenagers with high normative beliefs about aggression are more likely to become bullies and victims of traditional bullying and cyberbullying (Burton et al., 2013). Finally, normative beliefs about aggression can significantly predict the support and reinforcement of bystanders in offline bullying and cyberbullying (Machackova and Pfetsch, 2016).

According to Bandura’s social cognitive theory (Bandura, 1989), violent video games can initiate adolescents’ observational learning. In this situation, not only can they imitate the aggressive behavior of the model but also their understanding and acceptability about aggression may change. Therefore, normative beliefs about aggression can also be a mediator between violent video games and adolescent aggression (Duan et al., 2014; Anderson et al., 2017; Huesmann et al., 2017). Studies have shown that the mediating role of normative beliefs about aggression is not influenced by factors such as gender, prior aggression, and parental monitoring (Gentile et al., 2014).

### Family Environment, Violence Video Games, and Aggression

Family violence, parenting style, and other family factors have major effects on adolescent aggression. On the one hand, family environment can influence directly on aggression by shaping adolescents’ cognition and setting up behavioral models. Many studies have found that family violence and other negative factors are positively related to adolescent aggression (Ferguson et al., 2009, 2012; Ferguson, 2013), while active family environment can reduce the aggressive behavior (Batanova and Loukas, 2014).

On the other hand, family environment can act on adolescent aggression together with other factors, such as exposure to violent video games. Analysis of the interaction between family conflict and media violence (including violence on TV and in video games) to adolescent aggression showed that teenagers living in higher conflict families with more media violence exposure show more aggressive behavior (Fikkers et al., 2013). Parental monitoring is significantly correlated with reduced media violence exposure and a reduction in aggressive behavior 6 months later (Gentile et al., 2014). Parental mediation can moderate the relationship between media violence exposure and normative beliefs about aggression, i.e., for children with less parental mediation, predictability of violent media exposure on normative beliefs about aggression is stronger (Linder and Werner, 2012). Parental mediation is closely linked to decreased aggression caused by violent media (Nathanson, 1999; Rasmussen, 2014; Padilla-Walker et al., 2016). Further studies have shown that the autonomy-supportive restrictive mediation of parents is related to a reduction in current aggressive behavior by decreasing media violence exposure; conversely, inconsistent restrictive mediation is associated with an increase of current aggressive behavior by enhancing media violence exposure (Fikkers et al., 2017).

### The Current Study

Despite GAM and CM hold opposite views on the relationship between violent video games and aggression, both of the two models imply the same idea that aggression cannot be separated from internal and external factors. While emphasizing on negative effects of violent video games on adolescents’ behavior, the GAM uses internal factors to explain the influencing mechanism, including aggressive beliefs, aggressive behavior scripts, and aggressive personality (Bushman and Anderson, 2002; Anderson and Carnagey, 2014). Although the CM considers that there is no significant relation between violent video games and aggression, it also acknowledges the role of external factors such as violent video games and family violence. Thus, these two models seem to be contradictory, but in fact, they reveal the mechanism of aggression from different points of view. It will be more helpful to explore the effect of violent video games on aggression from the perspective of combination of internal and external factors.

Although previous studies have investigated the roles of normative beliefs about aggression and family factors in the relationship between violent video games and adolescent aggression separately, the combined effect of these two factors remains unstudied. The purpose of this study was to analyze the combined effect of normative beliefs about aggression and family environment. This can not only confirm the effects of violent video games on adolescent aggression further but also can clarify the influencing mechanism from the integration of GAM and CM to a certain extent. Based on the above, the following three hypotheses were proposed:

Hypothesis 1: There is a significant positive correlation between exposure to violent video games and adolescent aggression.
Hypothesis 2: Normative beliefs about aggression are the mediator of exposure to violent video games and adolescent aggression.
Hypothesis 3: The family environment can moderate the mediation effects of normative beliefs about aggression in exposure to violent video games and adolescent aggression; exposure to violent video games, family environment, normative beliefs about aggression, and aggression constitute a moderated mediation model.

## Materials and Methods

### Participants

All subjects gave informed written consent for participation in this investigation, and their parents signed parental written informed consent. The study was reviewed and approved by the Professor Committee of School of Psychology, Nanjing Normal University, which is the committee responsible for providing ethics approvals. A total of 648 Chinese middle school students participated in this study, including 339 boys and 309 girls; 419 students were from cities and towns, and 229 from the countryside. There were 277 and 371 junior and high school students, respectively. Ages ranged from 12 to 19 years, averaging 14.73 (*SD* = 1.60).

### Measures

#### Video Game Questionnaire (VGQ)

The Video Game Questionnaire (Anderson and Dill, 2000) required participants to list their favorite five video games and assess their use frequencies, the degree of violent content, and the degree of violent images on a 7-point scale (1, participants seldom play video games, with no violent content or image; 7, participants often play video games with many violent contents and images). Methods for calculating the score of exposure to violent video games: (score of violent content in the game + score of violent images in the game) × use frequency/5. Chen et al. (2012) found that the Chinese version of this questionnaire had high internal consistency reliability and good content validity. The Chinese version was used in this study, and the Cronbach’s α coefficient of the questionnaire was 0.88.

#### Aggression Questionnaire (AQ)

There were 29 items in AQ (Buss and Perry, 1992), including four dimensions: physical aggression, verbal aggression, anger, and hostility. The scale used 5-point scoring criteria (1, very incongruent with my features; 5, very congruent with my features). Scores for each item were added to obtain the dimension score, and dimension scores were summed to obtain the total score. The Chinese version of AQ had good internal consistency reliability and construct validity (Ying and Dai, 2008). In this study, the Chinese version was used and its Cronbach’s α coefficient was 0.83.

#### Family Environment Scale (FES)

The FES (Moos, 1990) includes 90 true-false questions and is divided into 10 subscales, including cohesion, expressiveness, conflict, independence, achievement-orientation, intellectual-cultural orientation, active-recreational orientation, moral-religious emphasis, organization, and control. The Chinese version of FES was revised by Fei et al. (1991) and used in this study. Three subscales closely related to aggression were selected, including cohesion, conflict, and moral-religious emphasis, with 27 items in total. The family environment score was the sum of scores of these three subscales (the conflict subscale was first inverted). The Cronbach’s α coefficient of the questionnaire was 0.75.

#### Normative Beliefs About Aggression Scale (NOBAGS)

There are 20 items in the NOBAGS (Huesmann and Guerra, 1997), which includes retaliation (12 items) and general (8 items) aggression belief. A 4-point Likert scale is used (1, absolutely wrong; 4, absolutely right). The subjects were asked to assess the accuracy of the behavior described in each item. High score means high level of normative beliefs about aggression. The revised Chinese version of NOBAGS consists of two factors: retaliation (nine items) and general (six items) aggression belief. Its internal consistency coefficient and test-retest reliability are 0.81 and 0.79. Confirmative factor analysis showed that this version has good construct validity: χ^2^ = 280.09, *df* = 89, χ^2^/*df* = 3.15, RMSEA = 0.07, SRMR = 0.04, NFI = 0.95, NNFI = 0.96, and CFI = 0.96 (Shao and Wang, 2017). In this study, the Cronbach’s α coefficient of the Chinese version was 0.88.

### Procedures

Group testing was performed in randomly selected classes of six middle schools. All subjects completed the above four questionnaires.

### Data Analysis

IBM SPSS Statistics 22 was used to analysis the correlations among study variables, the mediating effect of normative beliefs about aggression on the relationship between exposure to violent video games and aggression, and the moderating role of family environment in the relationship between exposure to violent video games and normative beliefs about aggression. In order to validate the moderated mediation model, Mplus 7 was also used.

## Results

### Correlation Analysis Among Study Variables

In this study, self-reported questionnaires were used to collect data, and results might be influenced by common method bias. Therefore, the Harman’s single-factor test was used to assess common method bias before data analysis. The results showed that eigenvalues of 34 unrotated factors were greater than 1, and the amount of variation explained by the first factor was 10.01%, which is much less than 40% of the critical value. Accordingly, common method bias was not significant in this study.

As described in Table 1, the degree of exposure to violent video games showed significant positive correlations to normative beliefs about aggression and aggression; family environment was negatively correlated to normative beliefs about aggression and aggression; normative beliefs about aggression were significantly and positively related to aggression. The gender difference of exposure to violent video games (*t* = 7.93, *p* < 0.001) and normative beliefs about aggression (*t* = 2.74, *p* < 0.01) were significant, which boys scored significantly higher than girls.

**Table 1:** Means, standard deviations, and Pearson correlations among study variables.

	Mean (*SD*)	1	2	3	4
1. Violent video exposure	11.13 (10.54)	1			
2. Family environment	18.82 (4.06)	−0.09^*^	1		
3. Normative beliefs about aggression	32.49 (8.89)	0.20^***^	−0.34^***^	1	
4. Aggression	74.70 (16.04)	0.26^***^	−0.29^***^	0.34^***^	1

* *p* < 0.05,

*** *p* < 0.001.

### Mediating Effect Analysis

To examine the mediation effect of normative beliefs about aggression on the relationship between exposure to violent video games and aggression, gender factor was controlled firstly. Stepwise regression analysis showed that the regression of aggression to violent video games (*c* = 0.28, *t* = 6.96, *p* < 0.001), the regression of normative beliefs about aggression to violent video games (*a* = 0.19, *t* = 4.69, *p* < 0.001), and the regression of aggression to violent video games (*c*′ = 0.22, *t* = 5.69, *p* < 0.001) and normative beliefs about aggression (*b* = 0.31, *t* = 8.25, *p* < 0.001) were all significant. Thus, normative beliefs about aggression played a partial mediating role in exposure to violent video games and aggression. The mediation effect value was 0.06, accounting for 21.43% (0.06/0.28) of the total effect.

### Moderated Mediation Effect Analysis

After standardizing scores of exposure to violent videogames, normative beliefs about aggression, family environment, and aggression, two interaction terms were calculated, including family environment × exposure to violent video games and family environment × normative beliefs about aggression. Regression analysis was carried out after controlling gender factor (Table 2).

**Table 2:** Moderated mediation effect analysis of the relationship between violent video exposure and aggression.

	Model 1 (criterion: AG)				Model 2 (criterion: NBA)				Model 3 (criterion: AG)			
	*B*	*SE*	*β*	95% CI	*B*	*SE*	*β*	95% CI	*B*	*SE*	*β*	95% CI
V VE	0.24	0.04	0.24^***^	[0.16, 0.31]	0.13	0.04	0.13^**^	[0.06, 0.21]	0.21	0.04	0.21	[0.13, 0.28]
FE	−0.27	0.04	−0.27^***^	[−0.34, −0.20]	−0.32	0.04	−0.32^***^	[−0.39, −0.25]	−0.19	0.04	−0.19^***^	[−0.27, −0.12]
V VE × FE	−0.04	0.03	−0.05	[−0.11, 0.02]	−0.12	0.03	−0.13^***^	[−0.19, −0.06]	−0.02	0.04	0.02	[−0.09, 0.05]
NBA									0.24	0.04	0.24^***^	[0.16, 0.32]
NBA × FE									0.02	0.04	0.02	[−0.06, 0.19]
Gender	0.05	0.08	0.03	[−0.10, 0.20]	−0.16	0.08	−0.08^*^	[−0.31, −0.01]	0.09	0.08	0.05	[−0.06, 0.24]
*R* ^2^	0.14				0.17				0.19			
*F*	27.12^***^				31.80^***^				25.38^***^			

V VE, violent video exposure; FE, family environment; NBA, normative beliefs about aggression; AG, aggression.

* *p* < 0.05,

** *p* < 0.01,

*** *p* < 0.001.

In the first step, a simple moderated model (Model 1) between exposure to violent video games and aggression was established. The result showed that exposure to violent video games had a significant effect on aggression (*c*_1_ = 0.24, *t* = 6.13, *p* < 0.001), while the effect of family environment × exposure to violent video games on aggression was not significant (*c*_3_ = 0.05, *t* = −1.31, *p* = 0.19), indicating that the relationship between exposure to violent video games and aggression was not moderated by family environment.

Next, a moderated model (Model 2) between exposure to violent video games and normative beliefs about aggression was established. The results showed that exposure to violent video games had a significant effect on normative beliefs about aggression (*a*_1_ = 0.13, *t* = 3.42, *p* < 0.001), and the effect of family environment × exposure to violent video games on normative beliefs about aggression was significant (*a*_3_ = −0.13, *t* = −3.63, *p* < 0.01).

In the third step, a moderated mediation model (Model 3) between exposure to violent video games and aggression was established. As shown in Table 2, the effect of normative beliefs about aggression on aggression was significant (*b*_1_ = 0.24, *t* = 6.15, *p* < 0.001), and the effect of family environment × exposure to violent video games on normative beliefs about aggression was not significant (*b*_2_ = 0.02, *t* = 0.40, *p* = 0.69). Because both *a*_3_ and *b*_1_ were significant, exposure to violent video games, family environment, normative beliefs about aggression, and aggression constituted a moderated mediation model. Normative beliefs about aggression played a mediating role between exposure to violent video games and aggression, while family environment was a moderator between exposure to violent video games and normative beliefs about aggression. Mplus analysis proved that the moderated mediation model had good model fitting (χ^2^/*df* = 1.54, CFI = 0.99, TLI = 0.98, RMSEA = 0.03, and SRMR = 0.01).

**Figure 1:**
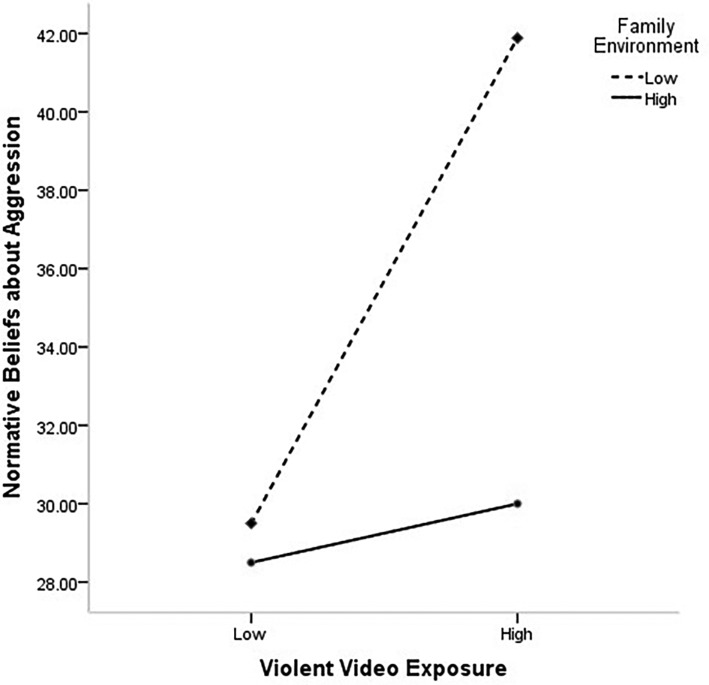
The moderating effect of the family environment on the relationship between violent video game exposure and normative beliefs about aggression.

To further analyze the moderating effect of the family environment and exposure to violent video games on normative beliefs about aggression, the family environment was divided into the high and low groups, according to the principle of standard deviation, and a simple slope test was performed (Figure 1). The results found that for individuals with high score of family environment, prediction of exposure to violent video games to normative beliefs about aggression was not significant (*b* = 0.08, *SE* = 0.08, *p* = 0.37). For individuals with low score of family environment, exposure to violent video games could significantly predict normative beliefs about aggression (*b* = 0.34, *SE* = 0.09, *p* < 0.001). Based on the overall findings, individuals with high scores of family environment showed a nonsignificant mediating effect of normative beliefs about aggression on the relation of exposure to violent video games and aggression; however, for individuals with low scores of family environment, normative beliefs about aggression played a partial mediating role in the effect of exposure to violent video games on aggression.

## Discussion

### Main Findings and Implications

This study found a significantly positive correlation between exposure to violent video games and adolescent aggression, corroborating existing studies (Anderson, 2004; Anderson et al., 2010; DeLisi et al., 2013; Greitemeyer and Mugge, 2014). Anderson et al. (2017) assessed teenagers in Australia, China, Germany, the United States, and other three countries and found that exposure to violent media, including television, movies, and video games, is positively related to adolescent aggression, demonstrating cross-cultural consistency; 8% of variance in aggression could be independently explained by exposure to violent media. In this study, after controlling for gender and family environment, *R* ^2^ for exposure to violent video games in predicting adolescent aggression was 0.05, indicating that 5% of variation in adolescent aggression could be explained by exposure to violent media. These consistent findings confirm the effect of exposure to violent video games on adolescent aggression and can be explained by the GAM. According to the GAM (Bushman and Anderson, 2002; Anderson and Carnagey, 2014), violent video games can make teenagers acquire, repeat, and reinforce aggression-related knowledge structures, including aggressive beliefs and attitude, aggressive perceptual schemata, aggressive expectation schemata, aggressive behavior scripts, and aggression desensitization. Therefore, aggressive personality is promoted, increasing the possibility of aggressive behavior. The Hypothesis 1 of this study was validated and provided evidence for the GAM.

As shown above, normative beliefs about aggression had a partial mediation effect on the relationship between exposure to violent video games and aggression. Exposure to violent video games, on the one hand, can predict adolescent aggression directly; on the other hand, it had an indirect effect on adolescent aggression *via* normative beliefs about aggression. According to the above results, when exposure to violent video games changes by 1 standard deviation, adolescent aggression varies by 0.28 standard deviation, with 0.22 standard deviation being a direct effect of exposure to violent video games on adolescent aggression and 0.06 standard deviation representing the effect through normative beliefs about aggression. Too much violence in video games makes it easy for individuals to become accustomed to violence and emotionally apathetic towards the harmful consequences of violence. Moreover, it can make individuals accept the idea that violence is a good way of problem solving, leading to an increase in normative beliefs about aggression; under certain situational cues, it is more likely to become violent or aggressive. This conclusion is supported by other studies (Gentile et al., 2014; Anderson et al., 2017; Huesmann et al., 2017). Like Hypothesis 1, Hypothesis 2 was validated the GAM.

One of the main findings of this study was the validation of Hypothesis 3: a moderated mediation model was constructed involving exposure to violent video games, family environment, normative beliefs about aggression, and aggression. Family environment moderated the first half of the mediation process of violent video games, normative beliefs about aggression, and aggression. In this study, family environment encompassed three factors, including (1) cohesion reflecting the degree of mutual commitment, assistance, and support among family members; (2) conflict reflecting the extent of anger, aggression, and conflict among family members; and (3) moral-religious emphasis reflecting the degree of emphasis on ethics, religion, and values. Individuals with high scores of family environment often help each other; seldom show anger, attack, and contradiction openly; and pay more attention to morality and values. These positive aspects would help them understand violence in video games from the right perspective, reduce recognition and acceptance of violence or aggression, and diminish the effect of violent video games on normative beliefs about aggression. Hence, exposure to violent video games could not predict normative beliefs about aggression of these individuals. By contrast, individuals with low scores of family environment are less likely to help each other; they often openly show anger, attack, and contradiction and do not pay much attention to morality and values. These negative aspects would not decrease but increase their acceptance of violence and aggression. For these individuals, because of the lack of mitigation mechanisms, exposure to violent video games could predict normative beliefs about aggression significantly.

The moderated mediation model of the relationship between exposure to violent video games and aggression could not only help reveal that exposure to violent video games can affect aggression but also provide an elaboration of the influencing mechanism. According to this model, for individuals with high scores of family environment, exposure to violent video games had only direct effect on aggression. However, for those with low scores of family environment, there was not only a direct effect of exposure to violent video games on aggression but also an indirect effect mediated by normative beliefs about aggression. In short, exposure to violence video games affecting aggression through normative beliefs about aggression is more likely to happen to adolescents with poor family environment than those with good family environment. That is, generation of adolescent aggression is not only related to internal cognitive factors but also to external situations. As Piotrowski and Valkenburg (Piotrowski and Valkenburg, 2015; Valkenburg, 2015) pointed out, the effect of violent video games/media on adolescents is a complex interaction of dispositional, developmental, and social factors, and individual differences in susceptibility to these three factors determine the nature and the extent of this influence. The proposed model incorporated some perspectives of GAM and CM: while confirming the effect of exposure to violent video games on aggression occurrence, the combined effect of individual and environmental factors was verified.

Compared with the simple mediation or moderation model, the present moderated mediation model provided deeper insights into the internal mechanism of the effect of violent video games on aggression, providing inspirations for preventing adolescent aggression. First, in view of the close relationship between exposure to violent video games and adolescent aggression, relevant government departments should continue to improve the grading system of video games; meanwhile, parents should appropriately monitor the types of video games used by teenagers as well as the time spent and reduce the degree of exposure to violent video games. Second, by allowing teenagers to objectively distinguish between violence in games and reality, the mediating role of normative beliefs about aggression could inspire people to identify rational ways to solve violence problems and to experience the hurtful consequences of aggression. This would help adolescents change normative beliefs about aggression, establish a correct view of right and wrong, and reduce the occurrence of aggression. Finally, the moderating effect of family environment on the mediation process suggests that more attention should be paid to the important role of family environment. On the one hand, family education is closely related to adolescent aggression. Then, parents should create a good family atmosphere, publicly show anger and aggression as little as possible, and advocate and practice positive moral values. Parents should adopt authoritative styles, abandoning autocratic and indulgent parenting styles (Casas et al., 2006; Sandstrom, 2007; Underwood et al., 2009; Kawabata et al., 2011) to minimize the negative effect of exposure to violent video games. On the other hand, for teenagers with poor family environment, while reducing exposure to violent video games, it is particularly important to change their normative beliefs about aggression, no longer viewing aggression as an alternative way to solve problems.

### Limitations

Limitations of the current study should be mentioned. First, only Chinese school students were assessed, in a relatively small number, which could affect sample representativeness. A large sample of teenagers from different countries and in different ages, also including juvenile offenders, would be more accurate in revealing the effect of violent video games on adolescent aggression. Second, this study only focused on violent video games, not involving violent media such as internet and television, daily life events, wars, and other major social events. Indeed, these factors also have important effects on adolescent aggression, and their influencing mechanisms and combined effect are worth investigating further. Third, this study mainly adopted the self-report method. Use of peer, parent, or teacher reports to assess exposure to violent video games and aggression would help improve the effectiveness of the study. Fourth, there might be other mediators, moderating variables and relational models. In addition to normative beliefs about aggression and family environment, individual emotions, personality characteristics, school climate, and companions may play mediating or moderating roles in the relationship between violent video games and aggression. This study developed a moderated mediation model between family environment and normative beliefs about aggression, but the possibility of multiple mediation and mediated moderation models cannot be ruled out.

## Conclusion

The current study showed that exposure to violent video games is positively related to adolescent aggression; normative beliefs about aggression have a mediating effect on exposure to violent video games and adolescent aggression, while the family environment regulates the first part of the mediation process. For individuals with good family environment, exposure to violent video games only has a direct effect on aggression; however, for those with poor family environment, there is an indirect effect mediated by normative beliefs about aggression alongside a direct effect. This moderated mediation model incorporates some perspectives of GAM and CM, enriching studies of generative mechanism of adolescent aggression.

## Author Contributions

YW and RS conceived the idea of the study. RS analyzed the data. YW and RS interpreted the results and wrote the paper. YW discussed the results and revised the manuscript.

### Conflict of Interest Statement

The authors declare that the research was conducted in the absence of any commercial or financial relationships that could be construed as a potential conflict of interest.
